# Hydrogel Droplet Microarray for Genotyping Antimicrobial Resistance Determinants in *Neisseria gonorrhoeae* Isolates

**DOI:** 10.3390/polym13223889

**Published:** 2021-11-10

**Authors:** Boris Shaskolskiy, Ilya Kandinov, Dmitry Kravtsov, Alexandra Vinokurova, Sofya Gorshkova, Marina Filippova, Alexey Kubanov, Victoria Solomka, Dmitry Deryabin, Ekaterina Dementieva, Dmitry Gryadunov

**Affiliations:** 1 Center for Precision Genome Editing and Genetic Technologies for Biomedicine, Engelhardt Institute of Molecular Biology, Russian Academy of Sciences, 119991 Moscow, Russia; Ilya9622@gmail.com (I.K.); solo13.37@yandex.ru (D.K.); al.vinokurova14@gmail.com (A.V.); sonyagorshkova@gmail.com (S.G.); mafilippova@mail.ru (M.F.); kdem@biochip.ru (E.D.); grad@biochip.ru (D.G.); 2 State Research Center of Dermatovenerology and Cosmetology, Russian Ministry of Health, 107076 Moscow, Russia; alex@cnikvi.ru (A.K.); solomka@cnikvi.ru (V.S.); dgderyabin@yandex.ru (D.D.)

**Keywords:** hydrogels, oligonucleotide microarray, DNA hybridization, antimicrobial resistance determinants, mutations, *Neisseria gonorrhoeae*

## Abstract

A multiplex assay based on a low-density hydrogel microarray was developed to identify genomic substitutions in *N. gonorrhoeae* that determine resistance to the currently recommended treatment agents ceftriaxone and azithromycin and the previously used drugs penicillin, tetracycline, and ciprofloxacin. The microarray identifies 74 drug resistance determinants in the *N. gonorrhoeae* *penA*, *ponA*, *porB*, *gyrA*, *parC*, *rpsJ*, *mtrR*, *bla*_TEM_, *tetM*, and 23S rRNA genes. The hydrogel elements were formed by automated dispensing of nanoliter-volume droplets followed by UV-induced copolymerization of NH_2_-containing oligonucleotides with gel-forming monomers. Polybutylene terephthalate plates without special modifications were used as microarray substrates. Sequences and concentrations of immobilized oligonucleotides, gel composition, and hybridization conditions were carefully selected, and the median discrimination ratio ranged from 2.8 to 29.4, allowing unambiguous identification of single-nucleotide substitutions. The mutation identification results in a control sample of 180 *N. gonorrhoeae* isolates were completely consistent with the Sanger sequencing results. In total, 648 clinical *N. gonorrhoeae* isolates obtained in Russia during the last 5 years were analyzed and genotyped using these microarrays. The results allowed us to draw conclusions about the present situation with antimicrobial susceptibility of *N. gonorrhoeae* in Russia and demonstrated the possibility of using hydrogel microarrays to control the spread of antibiotic resistance.

## 1. Introduction

The steady increase in the resistance of *N. gonorrhoeae* to antimicrobials strongly impairs the efficacy of gonorrhea treatment and constitutes a significant clinical and public health challenge. Genotyping data associated with susceptibility to antimicrobial drugs are important both for surveillance of gonococcal resistance and for guiding individualized therapy of patients, especially considering the emergence of isolates with decreased susceptibility to ceftriaxone and azithromycin [1,2,3,4,5].

Since multiple mechanisms contribute to the resistance of *N. gonorrhoeae* to most antimicrobials, the identification of individual genetic determinants is insufficient to predict phenotypic resistance. More than 20 genomic loci containing approximately 100 potential mutations are associated with the resistance of *N. gonorrhoeae* to actual antimicrobials [6,7,8,9,10], a relatively large number of parameters to analyze by real-time PCR or Sanger sequencing. On the other hand, the number of parameters is rather small for whole-genome sequencing (WGS); moreover, the cost of WGS is still relatively high for routine use, and data processing and genome assembly are laborious procedures. Targeted gene sequencing based on the Ion AmpliSeq™ platform (Thermo Fischer Scientific, Waltham, MA, USA) or AmpliSeq for Illumina products can be used for accurate genotyping of antimicrobial resistance determinants. An alternative technology that has successfully been proven useful for simultaneous identification of multiple specific genomic targets is matrix oligonucleotide microarray analysis, which is currently widely used [11,12,13,14].

Surface functionalization is a key parameter that characterizes microarrays and determines the speed of analysis, the intensity of the positive signal, and the discrimination ratio (*I_p_/I_m_*) between perfect and imperfect hybridization duplexes. An innovative approach based on copper-free click immobilization of oligonucleotides by strain-promoted azide-alkyne cycloaddition between a dibenzocyclooctyne-functionalized probe and an azide-functionalized surface has been developed for planar microarrays [14]. This procedure allows the assembly of oligonucleotide complexes on the surface and efficient amplification reactions on a microarray platform. Another efficient approach is the use of various copolymers for surface modification, creating three-dimensional coatings, for example, silicon/silicon oxide slides coated with an *N,N*–dimethylacrylamide (DMA), *N,N*–acryloyloxysuccinimide (NAS), and 3-(trimethoxysilyl) propyl methacrylate (MAPS) copolymer [12,15,16].

A popular technique in microarray technology is the use of macroporous polymer monolithic layers to coat a substrate. The coatings were originally formed on the basis of the copolymer glycidyl methacrylate–*co*–ethylene dimethacrylate (GMA-EDMA); later, depending on the aim, the more hydrophilic glycerol dimethacrylate (GDMA) or more hydrophobic divinylbenzene were utilized as crosslinking agents [17,18,19]. Thus, by surface modification, various types of microarray substrates (hydrophilic, hydrophobic, differing in charge, etc.) can be obtained, allowing nucleic acid amplification and/or hybridization reactions.

Owing to their hydrophilic properties, polymer hydrogels are also preferred substrates for the immobilization of biomolecules. The traditional approach to microarray manufacturing is to coat a glass or plastic substrate with a uniform hydrogel layer prior to the application of probes or the synthesis of oligonucleotides in situ. Materials used for hydrogel substrates include polyethylene glycols and their derivatives, for example, polyethylene glycol diacrylates and polysaccharides (alginates) [20]. Three-dimensional microarrays have a much larger surface area than two-dimensional microarrays, which allows the immobilization of larger amounts of oligonucleotide probes and, therefore, the detection of higher signal intensities after the binding of labeled target biomolecules. Moreover, the pores of three-dimensional materials provide an aqueous environment, favoring the preservation of biomolecular conformations [21].

An original technology of low-density hydrogel microarrays was developed at the Engelhardt Institute of Molecular Biology (EIMB). On these microarrays, probes are immobilized in three-dimensional hydrogel elements in the form of hemispherical microdroplets attached to a flat substrate. In addition, a method of polymerization-driven immobilization of NH_2_-containing oligonucleotides in microarray gel droplets has been developed. In this method, a growing polymer chain contains reactive radicals and double bonds that readily react with active amino groups and incorporate them into the gel polymer structure. At alkaline pH, the unprotonated amino groups are activated and immobilized during methacrylamide gel polymerization with high efficiency (greater than 80%) due to their interaction with a bifunctional crosslinking agent (*N,N′*–methylenebisacrylamide). Methacrylamide is used as a gel-forming monomer instead of acrylamide since it is inactive in the nucleophilic addition of amino groups to its double bond. Nanoliter-volume drops of the polymerization mixture are applied to a substrate using needle pins of a mechanical robot. Robotic application of droplets of the polymerization mixture is characterized by high reproducibility of the gel element volume. The size of the gel elements depends on the robot pin diameter, surface hydrophobicity, and polymerization mixture viscosity. Ultraviolet (UV) irradiation is used for the joint polymerization of molecular probes (oligonucleotides, proteins, etc.) with the main components of the hydrogel and for the uniform incorporation of the immobilized compounds into the growing polymer structure. Uncured components of the reaction mixture are eluted from the gel during post-polymerization washing. For each microarray, the diameters of the gel elements and geometric deviations of the matrix from the given parameters are controlled using specialized optics and computerized image analysis. The proposed method for manufacturing hydrogel microarrays is fast, inexpensive, and suited for large–scale production.

Specialized low–density hydrogel microarrays with up to several hundred elements are successfully being used both for basic research and in clinical laboratory diagnostics, including the analysis of specific sequences of bacterial and viral genomes [22]. Previously, we developed a microarray to identify 12 causative agents of human reproductive tract infections, including *N. gonorrhoeae*, with simultaneous detection of numerous genetic drug resistance determinants [23]. We also developed a microarray to detect numerous mutations leading to the resistance of *N. gonorrhoeae* to ceftriaxone, a drug of choice for the treatment of gonorrhea [13].

The goal of this work was to develop and validate a latest-generation oligonucleotide hydrogel microarray with probes for detecting all known key mutations in *N. gonorrhoeae* leading to ceftriaxone, penicillin, tetracycline, ciprofloxacin, and azithromycin resistance that can be used to control the spread and antimicrobial resistance of the causative agent of gonococcal infection.

## 2. Materials and Methods

### 2.1. Collection and Characterization of N. gonorrhoeae Clinical Isolates

Clinical isolates of *N. gonorrhoeae* (648 samples) were collected by the State Research Center of Dermatovenerology and Cosmetology of the Russian Ministry of Health from different regions of the Russian Federation in 2016–2020. Samples were obtained by specialized medical organizations of dermatovenerological profiling from clinical specimens (urethral specimens from men and cervical/urethral specimens from women) of patients diagnosed with primary symptomatic uncomplicated gonorrhea. Each sample was collected from an individual patient. Sample collection, transportation, cultivation, and storage were performed according to a previously described protocol [24,25].

The minimum inhibitory concentrations (MICs) for ceftriaxone, benzylpenicillin, tetracycline, ciprofloxacin, and azithromycin were determined by the serial dilution method on chocolate agar. The breakpoints for susceptibility (S) and resistance (R) were established according to the European Committee on Antimicrobial Susceptibility Testing (EUCAST) guidelines [26]. DNA extraction was carried out using a Lytech kit (Moscow, Russia).

All isolates were characterized by NG-MAST typing using the standard NG-MAST protocol (https://pubmlst.org/bigsdb?db=pubmlst_neisseria_seqdef&page=schemeInfo&scheme_id=71, accessed on 10 August 2021). The variable internal regions of the *porB* and *tbpB* genes were amplified via PCR, and the subsequent products were purified and sequenced using the 3730xl Genetic Analyzer (Applied Biosystems, Tustin, CA, USA). Allele numbers for the *porB* and *tbpB* sequences and sequence types (STs) were assigned via the NG-MAST database. The characteristics of the samples are given in Table S1 (Supplementary Materials).

The list of analyzed samples and their characteristics including the NG-MAST types are given in Table S1 (Supplementary Materials).

### 2.2. Design of Oligonucleotide Probes for Microarray Immobilization and Primers for PCR Amplification

The genetic determinants associated with drug resistance in *N. gonorrhoeae* [6,7,8,27,28,29,30,31] to be studied using microarray analysis included the following:

- replacements Ala311→Val; Ile312→Met; Val316→Thr, Pro; Thr483→Ser; Ala501→Val, Thr, Pro; Asn512→Tyr; Gly542→Ser; Gly545→Ser and Pro551→Leu, Ser in mosaic and non-mosaic alleles of the *penA* gene encoding penicillin-binding protein 2 (PBP2)—resistance to cephalosporins (ceftriaxone);
- insertion of an aspartic acid codon at position 345 of the PBP2 protein (insAsp345) (*penA* gene)—decreased susceptibility to penicillins;
- substitution Leu421→Pro in penicillin-binding protein 1 (PBP1) (*ponA* gene)—resistance to penicillins;
- substitutions Gly120→Lys, Arg, Asp, Asn, Thr and Ala121→Asp, Asn, Gly, Val, Ser in the porin protein PorB (*porB* gene)—decrease in cell membrane permeability and resistance to penicillins, tetracyclines, and cephalosporins;
- plasmid β-lactamases and mutations Met182→Thr and Gly238→Ser—resistance to penicillins and likely emergence of resistance to cephalosporins;
- substitutions Ser91→Phe, Thr and Asp95→Asn, Gly, His, Tyr, Ala in DNA gyrase (*gyrA* gene)—resistance to fluoroquinolones;
- mutations in the promoter region of the *mtrR* gene (-35delA, -10insT, -10insTT)—overexpression of the MtrCDE efflux pump, resistance to penicillins, tetracyclines, macrolides, and cephalosporins;
- *tetM* plasmid—resistance to tetracyclines;
- substitution Val57→Met in ribosomal protein S10 (*rpsj* gene)—resistance to tetracyclines;
- replacements Ser87→Asn, Arg, Ile and Glu91→Gln, Gly, Lys, Ala in topoisomerase IV (*parC* gene)—resistance to fluoroquinolones;
- nucleotide substitutions A2058→G, C and A2059→G, C in 23S rRNA—resistance to azithromycin.

The discriminating oligonucleotide probes were designed using the Oligo Analyzer Tool software (Integrated DNA Technologies, https://www.idtdna.com/pages/tools/oligoanalyzer, accessed on 11 June 2021). The length and complexity of the analyzed sequence were considered—specifically, the presence of repeats and extended homopolymer sequences—to ensure the specificity of the probes for the analyzed sequence. For each position at which mutations were analyzed, a set of specific oligonucleotides capable of detecting known substitution variants was selected. OligoAnalyzer Tool software was used to calculate the melting temperature (T_m_) of each oligonucleotide, and, by varying the oligonucleotide length, the variation in T_m_ was assured to be no more than 2–3 °C. Oligonucleotides capable of forming hairpin-type secondary structures of the hairpin with a high T_m_ (free energy of structure formation calculated by the OligoAnalyzer Tool software ∆G ≤ –6 kcal/mol) were avoided. The position of the variable nucleotides to be detected was selected to be no further than 1–4 nucleotides from the middle of the discriminating probe.

**Figure 1:**
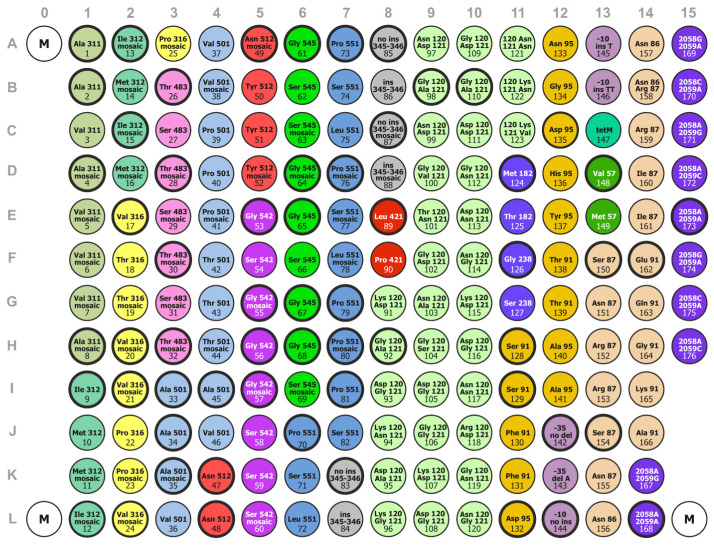
Configuration of the microarray for detecting mutations associated with drug resistance of *N. gonorrhoeae*. Circles representing elements containing probes with sequences that do not contain mutations have a bold outline. Element groups (highlighted in different colors): elements 1–8: Ala311→Val; elements 9–16: Ile312→Met; elements 17–25: Val316→Thr, Pro; elements 26–32: Thr483→Ser; elements 33–46: Ala501→Val, Thr, Pro; elements 47–52: Asn512→Tyr; elements 53–60: Gly542→Ser; elements 61–69: Gly545→Ser; elements 70–82: Pro551→Leu, Ser; elements 83–88: insAsp345—all in PBP2 (*penA* gene, mosaic and non-mosaic alleles); elements 89–90: Leu421→Pro in PBP1 (*ponA* gene); elements 91–123: Gly120→Lys, Arg, Asp, Asn, Thr; Ala121→Asp, Asn, Gly, Val, Ser in PorB (*porB* gene); elements 124–127: *bla*_TEM_ plasmid gene, Met182 (*bla*_TEM-1_)→Thr (*bla*_TEM-135_), Gly238→Ser (extended spectrum β–lactamase); elements 128–141: Ser91→Phe, Thr; Asp95→Asn, Gly, His, Tyr, Ala in DNA gyrase (*gyrA* gene); elements 142–146: -35delA, -10insT, and -10insTT in the *mtR* gene promoter region (efflux pump); element 147: *tetM* plasmid; elements 148–149: Val57→Met in ribosomal protein S10 (*rpsJ* gene); elements 150–166: Ser87→Asn, Arg, Ile; Glu91→Gln, Gly, Lys, Ala in topoisomerase IV (*parC* gene); elements 167–176: A2058→G, C; A2059→G, C in 23S rRNA.

The sequences of the designed probes are listed in Table S2 (Supplementary Materials). The nucleotide sequences of probes for analyzing mutations in the *penA* gene were designed considering the existence of different types of *penA* alleles: non-mosaic or mosaic. For example, in the group of probes (1–8) for analysis of mutations in codon 311, probes 1–3 corresponded to the sequences of non-mosaic *penA* alleles, and probes 4–8 corresponded to those of mosaic alleles with and without mutations (Table S2, Figure 1). In addition, when designing probes, we considered the characteristics of the Russian population of *N. gonorrhoeae* based on our previous experience in analyzing mutations in isolates collected in the Russian Federation [25]. For example, substitutions of residues 120–121 in the *porB* gene, which were most frequently found in the Russian isolates, were selected.

The amplification primers were designed on the basis of the *N. gonorrhoeae* genomic nucleotide sequences available from the National Center for Biotechnology Information (NCBI) database (http://www.ncbi.nlm.nih.gov, accessed on 10 June 2021). The primer sequences are listed in Table S3 (Supplementary Materials).

### 2.3. Synthesis of Oligonucleotide Probes and Primers for PCR

Oligonucleotide probes for microarray immobilization and primers for multiplex PCR were synthesized in a MerMade 48X automatic synthesizer (LGC Biosearch Technologies, Alexandria, MN, USA). The probes contained a spacer with a free amino group for covalent immobilization in the hydrogel; this spacer was introduced during synthesis using 5′-Amino-Modifier C6 (Glen Research, Sterling, VA, USA). Oligonucleotides were subjected to a two-step purification procedure including preparative polyacrylamide gel electrophoresis or ion exchange chromatography and semipreparative reversed–phase HPLC (Gilson, France). Quality control of the synthesized oligonucleotides was carried out by mass spectrometry.2.4. Microarray Design.

The microarray configuration—the layout of elements with immobilized oligonucleotide probes for identification of mutations associated with drug resistance of *N. gonorrhoeae*—is shown in Figure 1. The microarray elements in Figure 1 are presented as circles, with the element number and the determinants analyzed indicated inside. The element number corresponds to the immobilized probe number with the sequence shown in Table S1 (Supplementary Materials). The microarray consists of 179 hydrogel elements, including 176 elements with immobilized probes and three elements with a fluorescent marker (M, fluorescently labeled hexanucleotide 5′–NH_2_–AAATAT–NH-Cy5-3′) to allow correct positioning of the microarray when acquiring a fluorescence image after analysis. The elements are distributed into 19 groups based on the gene in which the presence of a mutation is analyzed. For example, the group of elements 1–8 is used to analyze mutations in residue 311 (Ala311→Val) in the PBP2 protein encoded by the *penA* gene; the group of elements 9–16 is used to analyze mutations in residue 312 (Ile312→Met) in PBP2, etc. The group distribution is shown in Figure 1, and the groups of elements are highlighted in different colors. Some groups also contain subgroups of elements for analysis of various determinants in one gene; for example, the group of elements 128–141 contains two subgroups for determining substitutions in codons 91 and 95 of the *gyrA* gene.

### 2.4. Microarray Manufacturing

Microarrays with oligonucleotides immobilized in a hydrogel were manufactured according to the following procedure. In this work, polybutylene terephthalate polymer plates were used as substrates. Each plate consisted of 16 clusters (Supplementary Materials Figure S1A). The composition for the preparation of copolymerization microarrays included 4.75% methacrylamide, 0.25% *N,N′*–methylenebisacrylamide, 50% glycerol, 5% *N,N,N′,N′*–tetramethylethylenediamine (TEMED) (all from Sigma-Aldrich, St. Louis, MO, USA), aqueous solutions of oligonucleotides at concentrations of 500–1000 pmol/μL, and Texas Red dye (0.1 pmol; Thermo Fischer Scientific, Waltham, MA, USA). The composition was stirred, transferred to the wells of a 384-well microtiter plate (Genetix, UK), and applied to polymer plates as an array of droplets using a modified QArray 2 high-throughput microarray spotter (Genetix, UK) with four pins 150 μm in diameter. The process of hydrogel droplet application by the robotic spotter is illustrated by the video file Video S1.avi (Supplementary Materials). Polymerization of gel elements was induced by UV irradiation with a wavelength of 312 nm and an intensity of 0.06 μV/cm^2^ for 45 min at 45 °C in a nitrogen flow. After the polymerization was complete, plates were sequentially washed with 0.1 M PBS buffer containing 0.1% Tween 20 (15 min, 22 °C), deionized water (15 min, 65 °C), and dried.

Polymer plates were cut into individual clusters using a laser fixed on a two-axis positioner. After cutting, the clusters were inserted into plastic holders of a microscope slide format and subjected to a technological quality control procedure. This stage included checking each microarray using a specialized fluorescence microscope equipped with a CCD camera and software (EIMB, Moscow, Russia) that recognized the microarray elements and calculated the main parameters (the diameter and geometric position of the elements in the matrix and the distance between the elements) prior to statistical processing and presenting information about the suitability of the microarray for further use. Thus, microarrays where the deviation in element diameters, element position, and the distance between elements exceeded 10% were discarded. For microarrays with a “positive” verdict, final assembly was carried out by mounting a composite hybridization chamber with a volume of 30 μL (Biochip-IMB, LLC, Moscow, Russia) (Figure S1B).

### 2.5. Multiplex PCR

Multiplex amplification and fluorescent labeling of the analyzed genome fragments were carried out in two separate test tubes with different primer mixtures: primer mixture 1 for amplification of *gyrA*, *parC*, *rpsJ*, 23S rRNA, *mtrR* (promoter region), *porB*, *ponA* and *tetM* and *bla*_TEM_ plasmid DNA and primer mixture 2 to amplify four segments of the *penA* gene (Table S3). The 30 μL reaction volume contained 3 U of HotStarTaq Plus DNA Polymerase, 1× Qiagen PCR buffer, 3 mm MgCl_2_, 200 μM each dNTP (all from Qiagen, Germany), 5 μM Sulfo-Cyanine 5 dUTP (Lumiprobe, Moscow, Russia), primer mixture 1 or 2, and 1 μL of the DNA template. The concentrations of primers in the reaction mixture are indicated in Supplementary Materials Table S3. For forward and reverse primers, two different universal adaptors were used. The reverse and forward adaptors were added at a ratio of 64:1 to obtain single-stranded PCR products. PCR was carried out in an S1000 thermal cycler (Bio-Rad Laboratories, Inc., Hercules, CA, USA) with the following sequential thermal cycling conditions: an initial denaturation step for 5 min at 95 °C; 29 cycles of denaturation at 95 °C for 30 s, annealing at 67 °C for 30 s, and extension at 72 °C for 30 s; 44 cycles of denaturation at 95 °C for 30 s, annealing at 54 °C for 30 s, and extension at 72 °C for 30 s; and a final extension step at 72 °C for 5 min. Finally, the PCR product mixture was cooled to 12 °C. The PCR products containing predominantly single-stranded, fluorescently labeled DNA fragments were combined from the two test-tubes, and 20 μL of the mixture was used for microarray hybridization.

### 2.6. Microarray Hybridization

Hybridization mixtures were prepared by mixing 10 μL of hybridization buffer (0.3 M HEPES (pH 7.5), 3.0 M guanidine thiocyanate (GuSCN), and 30 mm EDTA (all from Sigma-Aldrich, St. Louis, MO, USA) and 20 μL of the PCR product mixture. The microarray hybridization chambers were filled with the mixtures (30 μL), and the microarrays were incubated at 37 °C for 6–12 h. The hybridization chambers were then removed, and the microarray surfaces were washed three times with distilled water and air dried.

### 2.7. Detection and Interpretation of Fluorescence Signals

Acquisition of hybridization fluorescence patterns, measurement of signal intensities, and normalization were performed using a proprietary microarray scanner equipped with the original software [32]. The fluorescence signal of each microarray element was measured, and the minimum value of the fluorescence intensity was determined and set as the background signal intensity *I*_0_. The signal from element number N (*I_N_*) was considered positive if it was at least 5 times higher than the background signal (*I_N_*/*I*_0_ > 5) (see also Appendix A).

In each group (subgroup) of elements, the maximum positive signal *I_p_* and the next largest signal *I_m_* were determined. The discrimination ratio *I_p_/I_m_* was calculated and depending on the probe type in these elements (wild–type or mutant variant), the presence of a mutation in the analyzed locus was determined.

Box plots of the discrimination ratios in the groups of microarray elements were constructed using the ggplot2 software package for R (https://www.rdocumentation.org/packages/ggplot2, accessed on 30 August 2021).

## 3. Results

### 3.1. Selection of Optimal Conditions for DNA Hybridization on the Hydrogel Microarray

The important characteristics of an oligonucleotide microarray are its signal intensities and the discrimination ratio between perfect and imperfect duplexes *I_p_/I_m_*. These parameters are predetermined by the hydrogel composition, sequences and concentrations of the immobilized probes, and composition of the hybridization buffer.

#### 3.1.1. Selection of the Gel Composition

The gel composition is characterized by T (%), the ratio of the total mass of all gel-forming monomers to the volume of the gel; and C (%), the mass fraction of crosslinking monomers in relation to all monomers. An important factor affecting the hybridization time on hydrogel microarrays is retarded diffusion of DNA fragments into gel elements with immobilized oligonucleotides. To overcome the difficulties in diffusion, it is necessary to obtain a gel of sufficient porosity, which ensures free diffusion of the molecules of the analyzed samples. An increase in gel porosity can be achieved by reducing the concentration of gel-forming monomers (T). In addition, the pore size strongly depends on the concentration of the crosslinking agent (C).

The dependence of the fluorescence signal of microarray elements during hybridization on the monomer and crosslinking agent concentrations was further studied using *N. gonorrhoeae* 16S rRNA gene fragments of various lengths. The optimal gel composition was T5/C5 (Supplementary Materials Figure S2A). Lower concentrations of the monomer and crosslinking agent led to instability of the microarray elements during both polymerization and hybridization.

The gel composition significantly affected both the signal level and the *I_p_/I_m_* ratio in the transient hybridization mode, which determined the reliability of target detection using the hydrogel microarray. The hybridization time was estimated by monitoring the accumulation of the fluorescence signal in the microarray elements during hybridization (Supplementary Materials Figure S2B). A decrease in the amount of crosslinking agent allowed efficient hybridization of longer DNA fragments and significantly accelerated the diffusion of these fragments into the pores of the gel, reducing the overall hybridization time. Thus, an increase in the gel porosity led to the acceleration of hybridization kinetics, an increase in the signals, and an increase in the discrimination ratio. However, beginning at a certain threshold, a further increase in porosity led to deterioration of the mechanical properties (hydrogel instability).

#### 3.1.2. Oligonucleotide Probes and Hybridization Conditions

During microarray construction, the sequences of the oligonucleotide probes for immobilization were carefully selected (Section 2.2, Supplementary Materials Table S2). The lengths of the oligonucleotides, depending on the GC composition of the studied genome fragment, ranged from 15 to 23 nt, while the calculated T_m_ was 50–53 °C (in 1 M NaCl). Longer probe lengths generally led to a decrease in *I_p_/I_m_*.

Hybridization was carried out in a solution containing a buffer component to maintain pH, a salt to maintain ionic strength, and a chaotropic agent at a temperature depending on the melting point of the immobilized discriminating oligonucleotides. We previously showed that the use of 1 M GuSCN in the hybridization buffer lowered the melting temperatures of duplexes in the microarray elements by 8–10 °C on average [33], which allows hybridization at 37 °C. This temperature is convenient because most clinical laboratories are equipped with incubators to maintain this temperature.

The hybridization kinetics can significantly affect the *I_p_/I_m_* ratio in the initial (transient) kinetic mode, which in turn determines the reliability of the identification of specific targets using oligonucleotide microarrays. Since imperfect duplexes can form faster than perfect duplexes at the transient stage of hybridization [34], the *I_p_/I_m_* ratio is lower in the transient mode. Therefore, a short (30–60 min) hybridization time may be insufficient for reliable target identification on a hydrogel microarray. The saturation time can be shortened by decreasing the concentration of immobilized oligonucleotides or by changing the composition of the gel to increase its porosity. A 7–10-fold decrease in the concentration of immobilized oligonucleotides was shown to lead to an increase of at least twofold in the *I_p_/I_m_* ratio, with a 1.5–2.5-fold decrease in the total signal in the microarray elements during hybridization for 3–12 h [35]. Moreover, importantly, a total decrease in the concentration of all immobilized oligonucleotides could lead to a significant decrease in the signal below the detection threshold. This effect is particularly apparent for oligonucleotides, whose sequences can form high-melting-temperature secondary structures that prevent the formation of duplexes with the analyzed single-stranded fragment. Thus, the final concentrations of oligonucleotides in the hydrogel ranged from 50 to 100 pmol/μL, which was optimal for the positive signal values and the discrimination ratio *I_p_/I_m_*.

#### 3.1.3. Discrimination Ratio for Groups of Immobilized Oligonucleotides

**Figure 2:**
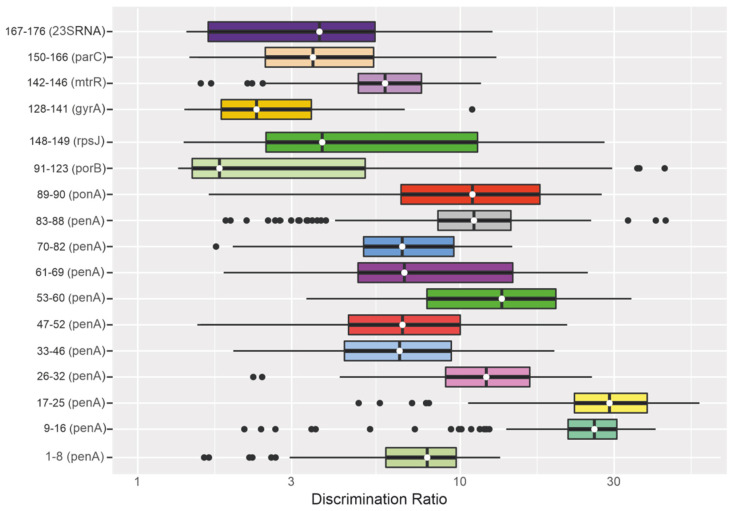
Box plot: Discrimination ratios in the groups of microarray elements, calculated as the ratio of the largest positive signal *I_p_* in the group to the next largest signal *I_m_*. The group numbers of microarray elements and the colors of the boxes correspond to those in Figure 1. Each box indicates the median *I_p_/I_m_* value for a group of elements, the lower (25th percentile) and upper (75th percentile) quartiles, the interquartile range, and data outliers (plotted as points). The whiskers extend to the boundaries of the interquartile range.

Our selection of the sequences and concentrations of immobilized oligonucleotides and the hydrogel composition made it possible to achieve optimal discrimination ratios between fluorescence signals in groups of elements for correct identification of point mutations. The *I_p_/I_m_* values were checked in a control sample of 180 *N. gonorrhoeae* isolates for which the presence of mutations was confirmed by Sanger sequencing. The results are presented in Figure 2 as a box plot.

Figure 2 does not include discrimination ratios for group 147 (the probe corresponding to the plasmid *tetM* gene) and group 124–127 (the probes corresponding to substitutions in the plasmid *bla*_TEM_ gene). When *tetM* plasmid DNA was detected, the signal of the corresponding microarray element was compared directly with the background signal, and if *I_p147_/I*_0_ > 5, it was concluded that *tetM* plasmid DNA was present. There were insufficient data for statistical analysis of the discrimination ratios for groups 124–127 since only two isolates in the control sample harbored the *bla*_TEM_ plasmid.

The median *I_p_/I_m_* value for all groups of probes ranged from 2.8 to 29.4, and the minimum *I_p_/I_m_* value was 1.5 in individual groups when single DNA samples were analyzed. Thus, the results could be unambiguously interpreted, and single-nucleotide substitutions could be detected in *N. gonorrhoeae* loci associated with resistance to antimicrobial drugs.

### 3.2. Detection of Mutations by Microarray Analysis

The steps for detecting mutations associated with antimicrobial resistance in *N. gonorrhoeae* using hydrogel droplet microarrays were as follows:

1. Multiplex amplification of fragments of the *N. gonorrhoeae* genome using specific primers with simultaneous fluorescent labeling;
2. Hybridization of fluorescently labeled PCR products to the microarray (Figure 1), with the formation of hybridization complexes in the microarray elements;
3. Detection of signals from the hybridization complexes and interpretation of the hybridization results.

**Figure 3:**
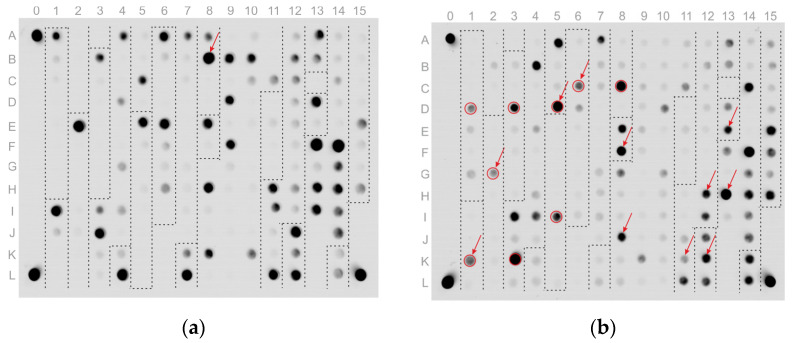
Fluorescence hybridization patterns of microarrays after analysis of DNA from *N. gonorrhoeae* isolates. Groups of elements (as denoted in Figure 1) are highlighted by dashed lines. Elements with detected mutations are noted with arrows, and elements with detected mosaic *penA* alleles are circled in red. (**a**) Isolate carrying the non-mosaic *penA* gene encoding PBP2 with the insertion of Asp at position 345. (**b**) Isolate carrying the mosaic *penA* gene with different substitutions and multiple mutations in the *ponA, porB, gyrA, parC, rpsJ*, and *mtrR* (promoter region) genes.

Examples of fluorescence images of microarrays after analysis (hybridization patterns) are shown in Figure 3. Figure 3a shows an example analysis of DNA of *N. gonorrhoeae* isolates susceptible to ceftriaxone, penicillin, tetracycline, ciprofloxacin, and azithromycin. The insertion of an aspartic acid codon at position 345 in the *penA* gene was detected, but this mutation did not lead to an increase in penicillin resistance.

Figure 3b shows the analysis results for DNA of *N. gonorrhoeae* isolate carrying:

- the mosaic *penA* gene encoding PBP2 with the Ile312→Met, Val316→Thr, Asn512→Tyr and Gly545→Ser substitutions (maximal signals in elements D-1 (group 1–8), K-1 (group 8–16), G-2 (group 17–25), D-3 (group 26–32), K-3 (group 33–46), D-5 (group 47–52), I-5 (group 53–60), C-6 (group 61–69) and C-8 (group 83–88));
- Leu421→Pro substitution in the *ponA* gene (maximal signal in element F-8 (group 89–90));
- Gly120→Lys and Ala121→Asn substitutions in the *porB* gene (maximal signal in element J-8 (group 91–123));
- Ser91→Phe substitution in the *gyrA* gene (maximal signal in element K-11 (group 128–141, subgroup for position 91));
- Asp95→Ala substitution in the *gyrA* gene (maximal signal in element H-12 (group 128–141, subgroup for position 95));
- -35delA deletion in the promoter region of the *mtrR* gene (maximal signal in element K-12 (group 142–146));
- Val57→Met substitution in the *rpsJ* gene (maximal signal in element E-13 (group 148–149));
- Ser87→Arg substitution in the *parC* gene (maximal signal in element H-13 (group 150–166, subgroup for position 87)).

Thus, the isolate is susceptible to azithromycin and resistant to penicillin—mutations in the *ponA*, *porB*, and *mtrR* (promoter region) genes; resistant to tetracycline—mutations in the *rpsJ*, *porB,* and *mtrR* (promoter region) genes; and resistant to ciprofloxacin—mutations in the *gyrA* and *parC* genes. A mosaic *penA* allele was identified with different substitutions that may be associated with resistance to third-generation cephalosporins. The isolate did show a decrease in susceptibility to ceftriaxone, and the measured MIC_cro_ = 0.12 mg/L was at the breakpoint between susceptibility and resistances, according to EUCAST guidelines: susceptible, MIC_cro_ ≤ 0.125 mg/L, resistant, MIC_cro_ > 0.125 mg/L.

The analytical sensitivity of the microarray was determined by assaying PCR products generated from serial dilutions of genomic DNA obtained from the isolate with the hybridization fluorescence pattern shown in Figure 3a (~200 genomic equivalents per reaction; 10^3^/16 dilution). The lowest dilution (10^3^/64 genomic equivalents per reaction; ~50 genomic copies) resulted in nonreproducible results due to stochastic effects during amplification. All thirteen PCR products were obtained in ~75% of the samples, whereas the other hybridization patterns had several low-intensity groups that could not be analyzed.

### 3.3. Analysis of Genetic Determinants of Antimicrobial Drug Resistance of N. gonorrhoeae in the Russian Population and Concordance of the Microarray Results with Phenotypic Data

**Figure 4:**
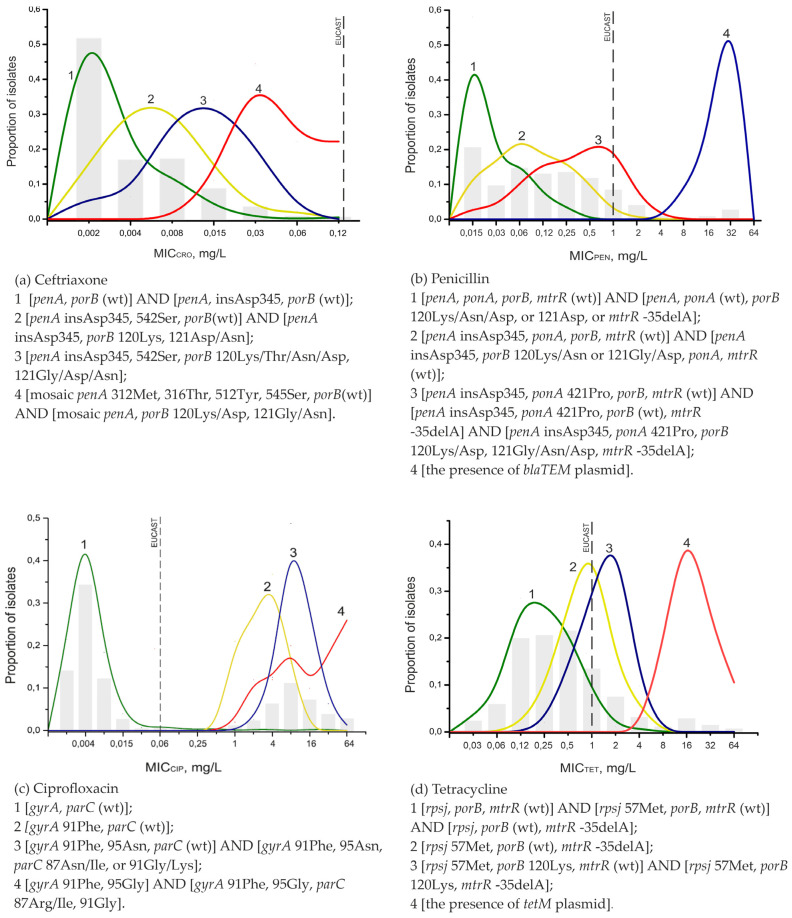
MIC distribution of *N. gonorrhoeae* isolates with different mutational profiles: ceftriaxone (**a**), penicillin (**b**), ciprofloxacin (**c**), tetracycline (**d**). The solid lines in the graphs indicate the proportion of isolates with a given MIC relative to the number of isolates with a given profile. The gray bars show the proportions of isolates with a given MIC relative to the total number of isolates. The vertical lines mark the breakpoints between susceptibility and resistance in isolates according to the EUCAST criteria. Abbreviation: wt—wild type.

Using the developed method, we analyzed 648 clinical isolates obtained in the Russian Federation in 2016–2020. The characteristics of the isolates are shown in Table S1. In total, collected isolates represented 238 different sequence types according to the NG-MAST analysis, with minimal clonal relatedness. Based on the results, mutation profiles of *N. gonorrhoeae* genes associated with susceptibility or resistance to penicillin, ceftriaxone, ciprofloxacin, and tetracycline were generated (Figure 4). Among the studied isolates, no azithromycin-resistant samples were found; for all samples, MIC_azm_ < 1.0 mg/L, and the A2058→G substitution in 23S rRNA was detected in only two samples.

All samples were phenotypically susceptible to ceftriaxone (MIC_cro_ < 0.125 mg/L), the drug of choice for the treatment of gonorrhea; however, a combination of mutations in the *penA*, *ponA*, and *porB* genes led to some increase in MIC_cro_, especially mutations in mosaic alleles of the *penA* gene: Ile312→Met, Val316→Thr, Asn512→Tyr, and Gly545→Ser (Figure 4a). Four samples were found with MIC_cro_ = 0.12 mg/L, which is the breakpoint between susceptibility and resistance according to the EUCAST guidelines; all of these carried the abovementioned mutations in mosaic *penA*. An example DNA analysis of such an isolate on a microarray is shown in Figure 3b.

Although penicillin, ciprofloxacin, and tetracycline have not been used in the Russian Federation for the treatment of gonorrhea since 2006, the proportion of *N. gonorrhoeae* isolates resistant to these drugs remains high. Changes in penicillin resistance are associated with the acquisition of mutations in the chromosomal genes *penA, ponA, porB*, and *mtrR.* Among the isolates, 8.5% of penicillin-resistant samples had MIC_pen_ > 1 mg/L; i.e., the trend toward a slight decrease in penicillin resistance, discovered in previous years (2005–2016) [24], still exists. As before, the most common determinant associated with penicillin resistance was insAsp345 (found in more than 70% of the isolates), and the second most common was the Leu421→Pro substitution in the *ponA* gene (36.6%). However, the single insAsp345 mutation did not always lead to a shift in MIC_pen_. The simultaneous presence of several mutations had a cumulative effect on the MIC_pen_ value (Figure 4b). The simultaneous presence of mutations in the *penA* and *ponA* genes and in four genes (profile 3 in Figure 4b) increased penicillin resistance to the level of resistant strains (MIC_pen_ > 1.0 mg/L). The presence of the bla_TEM_ plasmid caused an abrupt increase in the MIC_pen_ to 16–32 mg/L; this determinant was found in only 24 of the studied samples. Thus, the proportion of isolates possessing penicillinase plasmids with the gene encoding β-lactamase decreased from 5.6% to 3.7% over a five-year period.

Compared with previous data (2016), the proportion of isolates phenotypically resistant to ciprofloxacin remained similar (33.6%) [24]. Accordingly, the proportion of isolates carrying mutations in the *gyrA* and *parC* genes remained similar, 38.9%. Substitutions in these genes, including the single Ser91→Phe substitution in *gyrA*, resulted in a dramatic decrease in the susceptibility of *N. gonorrhoeae* to ciprofloxacin (Figure 4c).

The most commonly identified chromosomal mutations causing tetracycline resistance were the Val57→Met substitution in the *rpsJ* gene, mutations in the porin protein encoded by the *porB* gene, and mutations that increase the expression of the efflux pump (promoter region of the *mtrR* gene) (Figure 4d). The presence of the plasmid determinant *tetM* led to a shift in MIC_tet_ to 16–32 mg/L. The proportion of tetracycline-resistant isolates with MIC_tet_ ≥ 2 mg/L was 16.5%, the proportion of isolates carrying mutations in the *rpsJ* gene was 54%, and the proportion of isolates with the *tetM* determinant was 6%, which were also unchanged compared to those in 2016.

The -35delA mutation in the promoter region of the *mtrR* gene was found in 18% of isolates (notably, the insertion mutation -10insT (TT) was not present in any isolate). The most common substitutions in the porin protein were Gly120→Lys (11.4%) and Gly120→Asp (7%). Thus, the proportion of mutations in the genes affecting the influx and efflux of antimicrobials remained unchanged during the last five years.

## 4. Discussion

Molecular-based assays addressing the detection of antimicrobial resistance determinants became one of the standards and can offer advantages over phenotypic assays, such as multiplex targeting and more precise characterization of related genomic loci. PCR-based methods, especially digital PCR, are comparatively fast, convenient, and allow for simultaneous investigation of a large number of genetic markers of drug resistance. The further decrease in the cost of next-generation sequencing technologies and the subsequent expansion in bacterial WGS should provide simultaneous analysis of thousands of samples in routine clinical laboratory settings. The DNA microarrays for the detection and characterization of human pathogens including their antimicrobial resistance have also found their way into clinical practice in some countries [22]. The low-density arrays, in which individual elements detect specific sequence variations, provide limited and reliable diagnostic information. The key limitations of microarray-based assays are the use of modified glass slides as substrate and incomplete level of automation of all steps of the procedure that made the process costly and time-consuming [36].

In this study, we describe the development of a multiplex assay utilizing hybridization on low-density microarrays. The assay identifies *N. gonorrhoeae* genomic substitutions that determine resistance to drugs currently recommended for treatment (ceftriaxone and azithromycin) and to previously used drugs, such as penicillin, tetracycline, and ciprofloxacin. The core of the assay is a hydrogel droplet microarray with immobilized probes identifying 74 drug resistance determinants in the *penA*, *ponA*, *porB*, *gyrA*, *parC*, *rpsJ*, *mtrR*, *bla*_TEM_, *tetM*, and 23S rRNA genes.

Hydrogel elements are formed by automated dispensing of nanoliter-volume gel droplets followed by UV-induced copolymerization of NH_2_-containing oligonucleotides with gel-forming monomers. Via this process, an oligonucleotide probe forms a strong covalent bond with the polymer network, and the probes are uniformly distributed within a three-dimensional element (droplet) and are in a hydrophilic environment. To stabilize the droplets, 50% glycerol is added to the hydrogel–oligonucleotide mixture, which allows keeping the size and composition of the droplets unchanged. Such a viscous gel composition avoids evaporation both at the stage of droplet transfer to the substrates and at the subsequent stages of polymerization and washing. Thus, the concentrations of oligonucleotide probes in droplets remain constant throughout the entire batch of manufactured microarrays. The stability of the droplets is also ensured by the controlled maintenance of temperature and humidity in the spotter cabinet during application.

Each microarray was subjected to quality control using dedicated equipment and computerized image analysis. Microarrays with an intra-array variation in the drop dimension of at most 5% and an inter-array variation of less than 10% were selected for further analysis.

Different types of substrates, such as glass or plastic, can be used to prepare hydrogel microarrays. In this work, we used original polybutylene terephthalate plates, which, unlike glass substrates, do not require special modifications, for example, silanization. The plastic substrates and components of the microarray reaction chamber are mass produced by injection molding, which allows the cost of components to be reduced by an order of magnitude compared, for example, with ready-made commercial silane-coated slides.

Due to the three-dimensional configuration of the microarray elements, the total number of immobilized molecules is 2–3 orders of magnitude higher than that in surface microarrays, which results in much stronger fluorescence signals. Despite the slower hybridization kinetics in hydrogels, fluorescence signals emitted by the gel elements are 5–20 times higher than those emitted from the elements of surface microarrays, and the discrimination ratio between perfect and imperfect hybridization duplexes is 2–4 times higher [22]. Better discrimination crucially affects the development of diagnostic applications on hydrogel microarrays and is especially important for the identification of clinically relevant point mutations. The carefully selected gel composition, immobilized oligonucleotide sequences and concentration, and hybridization conditions allowed us to obtain median discrimination ratios ranging from 2.8 to 29.4, which made it possible to unambiguously identify single-nucleotide substitutions. In addition, high-intensity fluorescence signals from hydrogel microarrays can be acquired using simple, reliable, and inexpensive analyzers. In this work, an EIMB fluorescence analyzer, which can measure the signal intensity of microarray elements at three wavelengths from 380 to 850 nm with an accuracy of ±5% [32], was used to detect fluorescence signals.

The designed set of probes covers almost the full range of determinants of the resistance of *N. gonorrhoeae* to antimicrobial drugs that are currently the drugs of choice for the treatment of gonococcal infection. In addition, the microarray analysis included determinants of resistance to drugs previously used to treat gonorrhea but whose use was discontinued due to the emergence of a high level of resistance (penicillin, tetracycline, and ciprofloxacin). Modern control of gonococcal susceptibility to these drugs is important, first, for a detailed understanding of the spread of resistance mechanisms in the population, e.g., for monitoring changes in the frequency of resistance determinants after a drug has not been used for therapy for several years. Second, the increasing resistance of gonococci to the entire spectrum of antibiotics used dictates the need to identify new drugs, possibly based on previously used drugs.

The results of mutation identification in a control sample of 180 *N. gonorrhoeae* isolates were completely consistent with the results of Sanger sequencing. Furthermore, 648 clinical isolates obtained in the Russian Federation during the last 5 years (2016–2020) were analyzed and genotyped using microarrays. Generally, the presence of several mutations in *N. gonorrhoeae* had a cumulative effect on the MIC, indicating that the detection of one or several mutations is not sufficient to conclude that the isolate is resistant. Instead, a multiparametric analysis is required, and this ability is provided by the microarray developed herein.

The results obtained using microarrays allowed us to draw conclusions about the current situation regarding antimicrobial susceptibility and the presence of resistance determinants in *N. gonorrhoeae* isolates in the Russian Federation. The Russian *N. gonorrhoeae* population has remained stable over the past five years [37] with a decreasing trend in penicillin resistance. In addition, it remains susceptible to azithromycin, which is used worldwide. The proportion of isolates resistant to penicillin, tetracycline, and ciprofloxacin was greater than 5%, eliminating the hope that treatment of gonorrhea with these drugs could resume.

Notably, no cases of ineffective treatment of gonococcal infection with ceftriaxone have been recorded in Russia to date [13,24,25]. However, the identification of clinical isolates with mutations that reduce susceptibility to ceftriaxone emphasizes the need for continuous monitoring of *N. gonorrhoeae* antimicrobial susceptibility, particularly monitoring using the developed hydrogel droplet microarrays.

## Data Availability

The data presented in this study are available in the Supplementary Materials.

## Supplementary Materials

The following are available online at https://www.mdpi.com/article/10.3390/polym13223889/s1, Figure S1: Photograph of a polybutylene terephthalate plate with deposited clusters of hydrogel drops (A) and general arrangement drawing of the EIMB microarray with the composite hybridization chamber (B). Figure S2: Differences in fluorescence signal intensities and hybridization kinetics of fragments with different lengths in microarray elements with different gel compositions. Fluorescence hybridization patterns of DNA fragments 200, 500 and 1000 nt long in a microarray containing clusters of gel elements with different compositions (A). Signal accumulation curves in microarrays containing clusters of gel elements with different compositions (B). Video S1: application of hydrogel droplets on the polymer plates by the QArray 2 microarray spotter (Genetix, UK) using multiple pins. Table S1: Characteristics of *N. gonorrhoeae* clinical isolates collected in the Russian Federation and analyzed using microarrays. Table S2: Microarray elements according to the diagram in Figure 1, probe sequences and analyzed parameters. Table S3: Primers used for PCR amplification of N. gonorrhoeae gene fragments for microarray analysis.

## Appendix A

### On the Calculation of Background and Positive Signals of Microarray Elements

To correctly calculate the positive fluorescence signal values of the microarray elements during analysis of fluorescence images after hybridization, we used 180 fluorescence images obtained after analysis of *N. gonorrhoeae* samples with known mutations, which were previously characterized by Sanger sequencing. The microarray contains 176 elements. When processing the data, we distinguished 46 subgroups of elements, in which the “winner” is determined as the element with the highest of fluorescence signal intensity. Thus, the signals from the remaining 130 elements in each microarray could be considered insignificant (176 − 46 = 130).

Furthermore, the signal intensity values for all elements were normalized to the lowest value and ranked from lowest to highest by an order statistics approach. The average value for the 130th term in the variation series for the 180 fluorescence images was 4.34, and the upper boundary of the confidence interval at *p* = 0.999 was 5.06. The boundary of the confidence interval was used to determine positive fluorescence signals during analysis: for each fluorescence image of the microarray, the value of the minimum signal intensity (background signal, *I*_0_) was measured, and the signal of any element *I**_N_* for which *I**_N_*/*I*_0_ > 5 was considered positive.
