# Supplementary material for: Hydrogel Droplet Microarray for Genotyping Antimicrobial Resistance Determinants in Neisseria gonorrhoeae Isolates
Source: Polymers (Basel). 2021 Nov 10;13(22):3889. doi: 10.3390/polym13223889 (PMC8621812; doi:10.3390/polym13223889)
Supplement: Supplementary file 1 [file polymers-13-03889-s001.zip › Figure S1.pdf]

A

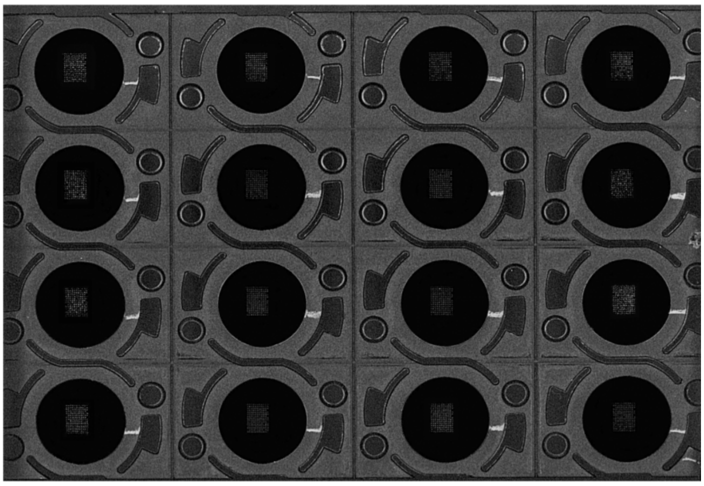

B

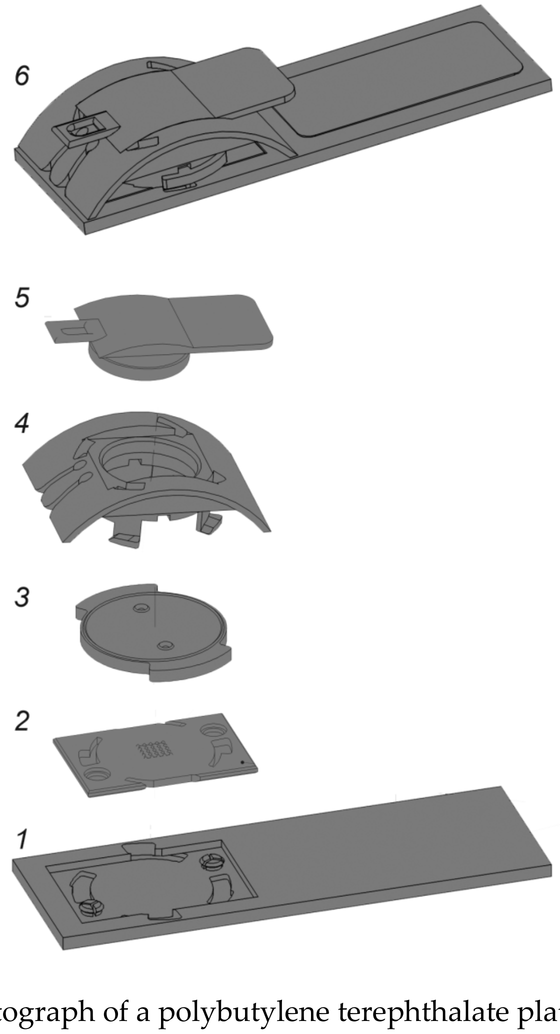

Figure S1. Photograph of a polybutylene terephthalate plate with deposited clusters of hydrogel microarrays, 16 clusters (A), and a general arrangement drawing of the EIMB microarray with the composite hybridization chamber (B).

1 – plastic holder for a cluster in a glass slide format;
2 - cluster with deposited hydrogel elements;
3 - clamping nut forming the volume of the reaction mixture (30 µl);
4 - hybridization chamber;
5 - elastic silicone cover;
6 - hydrogel microarray with assembled composite hybridization chamber.
