# Supplementary material for: Hydrogel Droplet Microarray for Genotyping Antimicrobial Resistance Determinants in Neisseria gonorrhoeae Isolates
Source: Polymers (Basel). 2021 Nov 10;13(22):3889. doi: 10.3390/polym13223889 (PMC8621812; doi:10.3390/polym13223889)
Supplement: Supplementary file 1 [file polymers-13-03889-s001.zip › Figure S2.pdf]

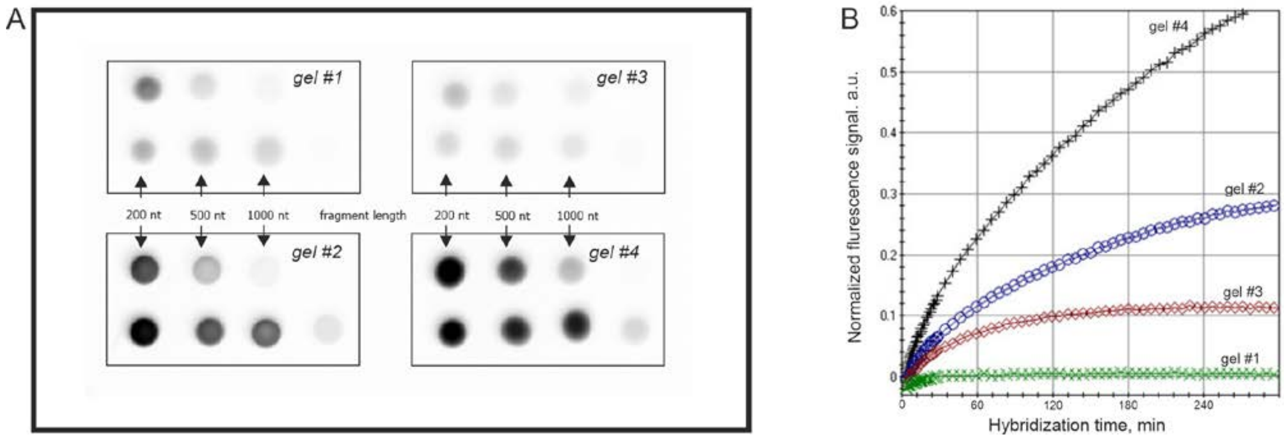


**Figure S2**. Dependence of fluorescent signals and kinetics of hybridization of fragments with different lengths in microarray elements differing in gel composition. A series of specialized microarrays differing in composition of the main components of the hydrogel T4/C25 (gel #1), T4/C5 (gel #2), T5/C25 (gel #3), T5/C5 (gel #4) (% of the main components) were manufactured by copolymerization technology as described in section 2.5; gel elements contained immobilized probes specific for the sequence of the *N. gonorrhoeae* 16S rRNA gene. Using primers specific to the 16S rRNA gene, DNA fragments of different lengths from 200 to 1000 nt were amplified and hybridized on a specialized microarray. (A) Fluorescence pattern of hybridization of DNA fragments 200, 500, 1000 nt long in microarray containing clusters of gel elements of different composition. (B) Signal accumulation curves in microarray containing clusters of gel elements of different composition.
