# Supplementary material for: Hydrogel Droplet Microarray for Genotyping Antimicrobial Resistance Determinants in Neisseria gonorrhoeae Isolates
Source: Polymers (Basel). 2021 Nov 10;13(22):3889. doi: 10.3390/polym13223889 (PMC8621812; doi:10.3390/polym13223889)
Supplement: Supplementary file 1 [file polymers-13-03889-s001.zip › Table S2.pdf]

Table S2. Biochip elements according to the scheme in Figure 1, probe sequences, and analyzed parameters

Element number	Group of elements, analyzed mutation	Amino acid	Probe sequence, 5'-3'	Drug-resistance
1	group 1-8: Ala-311→Val in PBP2 (*penA* gene)	Ala-311	CTGGTTCGGCAATCAAACC	Resistance to cephalosporins
2		Ala-311	CTGGTTCGGCCATCAAACC	
3		Val-311	CTGGTTCGGTCATCAAACC	
4		Ala-311 mosaic	CCGGTTCTGCCATGAAGCC	
5		Val-311 mosaic	CCGGTTCTGTCATGAAGCC	
6		Val-311 mosaic	CTGGTTCTGTCATGAAGCC	
7		Val-311 mosaic	CTGGTTCTGCCATGAAGCC	
8		Ala-311 mosaic	CCGGTTCGGCAATAAAGCC	
9	group 9-16: Ile-312→Met in PBP2 (*penA* gene)	Ile-312	TTCGGCAATCAAACCGTTCG	Resistance to cephalosporins
10		Met-312	TTCGGCAATGAAACCGTTCG	
11		Met-312 mosaic	GTTCTGCCATGAAGCCGTTTA	
12		Ile-312 mosaic	GTTCTGCCATCAAGCCGTTTA	
13		Ile-312 mosaic	GTTCGGCAATAAAGCCCTTTG	
14		Met-312 mosaic	GTTCTGTCATGAAGCCGTTTA	
15		Ile-312 mosaic	GTTCTGTCATCAAGCCGTTTA	
16		Met-312 mosaic	GTTCGGCAATGAAGCCCTTTG	
17	group 17-25: Val-316→Thr, Pro in PBP2 (*penA* gene)	Val-316	AACCGTTCGTGATTGCGAAG	Resistance to cephalosporins
18		Thr-316	AACCGTTCACCATTGCGAAG	
19		Thr-316 mosaic	AGCCGTTTACCATTGCCAAA	
20		Val-316 mosaic	AGCCGTTTGTCATTGCCAAA	
21		Val-316 mosaic	AGCCGTTTGTGATTGCCAAA	
22		Pro-316	AACCGTTCCCGATTGCGAAG	
23		Pro-316 mosaic	AGCCGTTTCCCATTGCCAAA	
24		Val-316 mosaic	AGCCCTTTGTCATTGCGAAA	
25		Pro-316 mosaic	AGCCCTTTCCCATTGCGAAA	
26	group 26-32: Thr-483→ Ser in PBP2 (*penA* gene)	Thr-483	GGCGGCACCGGTACG	Resistance to cephalosporins
27		Ser-483	GGCGGCAGCGGTACG	
28		Thr-483 mosaic	GGCGGTACCGGTACTG	
29		Ser-483 mosaic	GGCGGTAGCGGTACTG	
30		Thr-483	GGCGGTACGGGTACGG	

		mosaic		
31		Ser-483 mosaic	GGCGGTAGCGGTATTG	
32		Thr-483 mosaic	GGCGGTACGGGTATTG	
33	group 33-46: Ala-501→Val, Thr, Pro in PBP2 (*penA* gene)	Ala-501	GGTACGGCGCGCAAAT	Resistance to cephalosporins
34		Ala-501	GGCACGGCGCGCAAGT	
35		Ala-501 mosaic	GGTACGGCGCGTAAGT	
36		Val-501	GGTACGGTGCGCAAAT	
37		Val-501	GGCACGGTGCGCAAGT	
38		Val-501 mosaic	GGTACGGTGCGTAAGT	
39		Pro-501	GGTACGCCGCGCAAAT	
40		Pro-501	GGCACGCCGCGCAAGT	
41		Pro-501 mosaic	GGTACGCCGCGTAAGT	
42		Thr-501	GGTACGACGCGCAAAT	
43		Thr-501	GGCACGACGCGCAAGT	
44		Thr-501 mosaic	GGTACGACGCGTAAGT	
45		Ala-501	GGTACGGCGCGCAAAC	
46		Val-501	GGTACGGTGCGCAAAC	
47	group 47-52: Asn-512→Tyr in PBP2 (*penA* gene)	Asn-512	TATGTGGACAACAAACACGT	Resistance to cephalosporins
48		Asn-512	TATGCCGACAACAAACACGT	
49		Asn-512 mosaic	TACGTCGATAACAAACACGT	
50		Tyr-512	TATGTGGACTACAAACACGT	
51		Tyr-512	TATGCCGACTACAAACACGT	
52		Tyr-512 mosaic	TACGTCGATTACAAACACGT	
53	group 53-60: Gly-542→Ser in PBP2 (*penA* gene)	Gly-542	ACTGCCCACGGCTATTACG	Resistance to cephalosporins
54		Ser-542	ACTGCCCACAGCTATTACG	
55		Gly-542 mosaic	ACCGCCAACGGCTATTACG	
56		Gly-542	ACTGCCAACGGCTATTACG	
57		Gly-542 mosaic	GACTGCAAACGGTTACTAC	
58		Ser-542	ACCGCCAACAGCTATTACG	
59		Ser-542	ACTGCCAACAGCTATTACG	
60		Ser-542 mosaic	GACTGCAAACAGTTACTAC	
61	group 61-69: Gly-545→ Ser in PBP2 (*penA* gene)	Gly-545	GCTATTACGGCGGCGTAGT	Resistance to cephalosporins
62		Ser-545	GCTATTACAGCGGCGTAGT	
63		Ser-545 mosaic	GTTACTACAGCGGTGTAGT	
64		Gly-545 mosaic	GTTACTACGGCGGTGTAGT	
65		Gly-545	GCTATTACGGCGGCGTAGTG	
66		Ser-545	GCTATTACAGCGGCGTAGTG	
67		Gly-545	GTTACTACGGCGGCGTAGT	
68		Gly-545	GTTACTACGGCGGCGTAGTG	
69		Ser-545 mosaic	GTTACTACAGCGGCGTAGT	
70	group 70-82:	Pro-551	GGCAGGTCCGGTCTTCAAA	Resistance to

71	Pro-551→Leu, Ser in PBP2 (*penA* gene)	Ser-551	GGCAGGTTCGGTCTTCAAA	cephalosporins
72		Leu-551	GGCAGGTCTGGTCTTCAAA	
73		Pro-551	GGCAGGGCCGCCCTTCAAA	
74		Ser-551	GGCAGGGTCGCCCTTCAAA	
75		Leu-551	GGCAGGGCTGCCCTTCAAA	
76		Pro-551	GACAGGTCCGGTCTTCAAA	
77		Ser-551 mosaic	GACAGGTTCGGTCTTCAAA	
78		Leu-551 mosaic	GACAGGTCTGGTCTTCAAA	
79		Pro-551	GGCAGGTCCGCCCTTCAAA	
80		Pro-551 mosaic	GACAGGGCCGCCCTTCAAA	
81		Pro-551	GGCAGGGGCGCCCTTCAAA	
82		Ser-551	GACAGGGTCGCCCTTCAAA	
83	group 83-88: insAsp-(345-346) in PBP2 (*penA* gene)	No ins	CCGTGCGCGATACCCAT	Resistance to penicillins
84		insAsp-(345-346)	GTGCGCGATGATACCCAT	
85		No ins	CCGTGCGCGACACCCAT	
86		insAsp-(345-346)	GTGCGCGACGATACCCAT	
87		No ins mosaic	CCGTACAAGATACCCACGTT	
88		insAsp-(345-346) mosaic	GTACAAGATCATACCCAC	
89	group 89-90: Leu-421→Pro в PBP1 (*ponA* gene)	Leu-421	CCGTTGCTGCAGGGG	Resistance to penicillins
90		Pro-421	CCGTTGCCGCAGGG	
91	group 91-123: Gly-120→Lys, Arg, Asp, Asn, Thr; Ala-121→Asp, Asn, Gly, Val, Ser in PorB (*porB* gene)	Lys-120 Asp-121	ACACCAAGGACAACGTCAA	Resistance to penicillins, tetracyclines, cephalosporins
92		Gly-120 Ala-121	ACACCGGCGCCAACGTC	
93		Asp-120 Gly-121	ACACCGACGGCAACGTCAA	
94		Lys-120 Asn-121	ACACCAAGAACAACGTCAAT	
95		Asp-120 Ala-121	ACACCGACGCCAACGTCAA	
96		Lys-120 Gly-121	ACACCAAGGGCAACGTCAA	
97		Asn-120 Asp-121	ACACCAACGACAACGTCAA	
98		Gly-120 Ala-121	ACACCGGTGCCAACGTCAA	
99		Asn-120 Asp-121	AACACCAATGACAACGTCAAT	
100		Gly-120 Val-121	ACACCGGCGTCAACGTCAA	
101		Thr-120 Asn-121	ACACCACGAACAACGTCAA	
102		Gly-120 Asp-121	ACACCGGCGACAACGTCAA	
103		Asn-120 Ala-121	ACACCAACGCCAACGTCAA	
104		Gly-120 Ser-	ACACCAAGGACAACGTCAA	

		121		
105		Asp-120 Gly-121	ACACCGGCGCCAACGTC	
106		Gly-120 Gly-121	ACACCGACGGCAACGTCAA	
107		Lys-120 Asp-121	ACACCAAGAACAACGTCAAT	
108		Asp-120 Gly-121	ACACCGACGCCAACGTCAA	
109		Gly-120 Asp-121	ACACCAAGGGCAACGTCAA	
110		Gly-120 Ala-121	ACACCAACGACAACGTCAA	
111		Asp-120 Asp-121	ACACCGGTGCCAACGTCAA	
112		Gly-120 Asn-121	AACACCAATGACAACGTCAAT	
113		Asp-120 Asn-121	ACACCGGCGTCAACGTCAA	
114		Asn-120 Gly-121	ACACCACGAACAACGTCAA	
115		Lys-120 Asp-121	ACACCGGCGACAACGTCAA	
116		Asp-120 Gly-121	ACACCAACGCCAACGTCAA	
117		Asn-120 Asn-121	ACACCGGCGACAACGTCAA	
118		Arg-120 Asp-121	ACACCGGCGACAACGTCAA	
119		Gly-120 Asn-121	ACACCGGCGACAACGTCAA	
120		Asn-120 Gly-121	ACACCGGCGACAACGTCAA	
121		Asn-120 Asn-121	ACACCGGCGACAACGTCAA	
122		Lys-120 Asn-121	ACACCGGCGACAACGTCAA	
123		Lys-120 Val-121	ACACCGGCGACAACGTCAA	
124	group 124-127: bla_{TEM} plasmid, Met-182 (bla_{TEM-1} → Thr ($bla_{TEM-135}$); Gly-238→Ser (extended spectrum beta-lactamase)	Met-182	CACCACGATGCCTGCAGCA	Resistance to penicillins and cephalosporins
125		Thr -182	CACCACGACGCCTGCA	
126		Gly-238	TCTGGAGCCGGTGAGCGTG	
127		Ser-238	TCTGGAGCCAGTGAGCGTG	
128	group 128-141: Ser-91→Phe, Thr; Asp-95→Asn, Gly, His, Tyr, Ala in DNA-gyrase (*gyrA* gene)	Ser-91	GGCGATTCCGCAGTTTACGA	Resistance to fluoroquinolones
129		Ser-91	ACGGCGATTCCGCAGTTTA	
130		Phe-91	GGCGATTTCGCAGTTTACG	
131		Phe-91	ACGGCGATTTCGCAGTTTA	
132		Asp-95	GCAGTTTACGACACCATCGTC	
133		Asn-95	GCAGTTTACAACACCATCGTC	
134		Gly-95	CAGTTTACGGCACCATCGTC	
135		Asp-95	GCAGTTTACGATACCATCGTC	
136		His-95	GCAGTTTACCACACCATCGTC	
137		Tyr-95	GGCGATTTCGCAGTTTACGA	
138		Thr-91	CCACGGCGATTCCGCAGTTTAC	

139		Thr-91	CCACGGCGATTTCGCAGTTTAC	
140		Ala-95	CGCAGTTTACGACACCATCGTCC	
141		Ala-95	CGCAGTTTACAACACCATCGTCC	
142	group 142-146: -35 delA, -10 insT, -10 insTT in *mtR* gene, promoter region (efflux pump)	no del	ATTGCACGGATAAAAAGTCTTT	Resistance to penicillins, tetracyclines, macrolides, cephalosporins
143		-35 delA	ATTGCACGGATAAAAGTCTTT	
144		no ins	AAAGTCTTTTTTATAATCCGCCC	
145		10 insT	AAAGTCTTTTTTTATAATCCGCC	
146		10 insTT	AAAGTCTTTTTTTTATAATCCGCC	
147	group 147: tetM plasmid		TAGGAAAATGGGGATTCCCACAAT	Resistance to tetracyclines
148	group 148-149: Val-57→Met in ribosomal protein S10 (*rpsj* gene)	Val-57	TTCTCCGCACGTGAACAAAAC	Resistance to tetracyclines
149		Met-57	TTCTCCGCACATGAACAAAAC	
150	group 150-166: Ser-87→Asn, Arg, Ile; Glu-91→Gln, Gly, Lys, Ala in topoisomerase IV (*parC* gene)	Ser-87	GCGACAGTTCCGCCTATGA	Resistance to fluoroquinolones
151		Asn-87	GCGACAATTCCGCCTATGA	
152		Arg-87	GCGACCGTTCCGCCTATGA	
153		Arg-87	GCGACAGGTCCGCCTATGA	
154		Ser-87	ACGGCGACAGTTCCGCCT	
155		Asn-87	ACGGCGACAATTCCGCCT	
156		Asn-86	GCACGGCGACAGTTCCG	
157		Asn-86	GCACGGCAACAGTTCCG	
158		Asn-86 Arg-87	GCACGGCAACCGTTCCG	
159		Arg-87	ACGGCGACCGTTCCGCC	
160		Arg-87	GCGACATTTCCGCCTATGA	
161		Ile-87	ACGGCGACATTTCCGCCT	
162		Glu-91	CCTATGAGGCGATGGTGCG	
163		Gln-91	CCTATCAGGCGATGGTGCG	
164		Gly-91	CCTATGGGGCGATGGTGCG	
165		Lys-91	CCTATAAGGCGATGGTGCG	
166		Ala-91	CCTATGCGGCGATGGTGCG	
167	group 167-176: A2058→G,C; A2059→G,C in 23S rRNA	2059G	TAGACGGAGAGACCCCGTG	Resistance to macrolides
168		2058G	AGGACGGAAAGACCCCGTG	
169		2058G	AGGACGGGAAGACCCCGTG	
170		2058C	AGGACGGCAAGACCCCGTG	
171		2059G	AGGACGGAGAGACCCCGTG	
172		2059C	AGGACGGACAGACCCCGTG	
173		2058A 2059A	TAGACGGAAAGACCCCGTG	
174		2058G	TAGACGGGAAGACCCCGTG	
175		2058C	TAGACGGCAAGACCCCGTG	
176		2059C	TAGACGGACAGACCCCGTG	
M			Fluorescent marker	
