# Supplementary material for: Hydrogel Droplet Microarray for Genotyping Antimicrobial Resistance Determinants in Neisseria gonorrhoeae Isolates
Source: Polymers (Basel). 2021 Nov 10;13(22):3889. doi: 10.3390/polym13223889 (PMC8621812; doi:10.3390/polym13223889)
Supplement: Supplementary file 1 [file polymers-13-03889-s001.zip › Table S3.pdf]

Table S3. Primers for PCR amplification of *N. gonorrhoeae* gene fragments for microarray analysis

	Gene	Direction	Sequence, 5'-3'	Concentration in the reaction mixture (μM)	Amplicon length (bp)
1st set	*gyrA*	for	GCTGAAAAATAACTGGAATGCCGCCTACA	20.2	175
	gyrA	rev	ATCCGAAGTTGCCCTGTCCGTCTA	20.2	
	parC	for	GCATTTTGTTTGCCATGCGCGATATGG	23.6	157
	parC	rev	AGCGCAAGGTAAAATCCTGAGCCATG	23.6	
	rpsJ	for	CTGCACAAGAAATCGTTGAAACTGCAAA	26.9	218
	rpsJ	rev	GCCGGCAAATCCAGCTTCATCAGCG	26.9	
	23S rRNA	for	AAGGTAGCGAAATTCCTTGTCGG	33.7	338
	23S rRNA	rev	GGGAGGMGACCGCCCCAGTC	33.7	
	mtrR	for	CGAACGGGTTGCAAAGCAGATTATACCT	64.0	162
	mtrR	rev	GGTTTTTCTCATAATGGCGTTTTCGTTTCG	64.0	
	porB	for	GGGCAACAAACAATCCTTCGTC	33.7	195
	porB	rev	GGGAGAATCGTAGCGTRCGGACAGGTA	33.7	
	ponA	for	GGCCCGAGCGGTCGATAATGAGAAAAT	33.7	200
	ponA	rev	CGATTGAATGTTTTGCTGTGAAAATCA	33.7	
	bla_{TEM}	for	ACCGGAGCTGAATGAAGCCATACCAA	30.3	251
	bla_{TEM}	rev	CCCAGTGCTGCAATGATACCGC	30.3	
	tetM	for	CTGCAAAAGATGGCGTACAAGCACAAAC	30.3	112
	tetM	rev	TAAATCAATTCCATTTTGGTCAATCTTATT	30.3	
2nd set	*penA*	for	CCTACGATCCCAACAGACCCGGC	16.6	233
	penA	for	CCTATGAGCCCAACAAACCCGGTC	16.6	
	penA	rev	GCATAATGCCGCGCACATCCAAAG	16.6	
	penA	for	TATACCGCACTGACGCACGACGG	66.5	416
	penA	for	TATACTGTCTTGACCCATGACGG	66.5	
	penA	rev	RGTCAGDGGYTTGGTCGGGGAAA	66.5	
	penA	rev	GCGGGGGCAAAACCGATAAAGGTAG	41.5	268
	penA	rev	GCGGGGGCAAAACCGATAAACGTAC	41.5	
	penA	rev	GCCGGRGCAAAACCGATGAAAGTGG	41.5	
Universal adaptors		for	GGACGTGCGCAGG	167	-
		rev	AGTGCGGGACGGA	10670	-
